# Arbuscular mycorrhizal fungi modulate litter decomposition dynamics through alterations in stoichiometry, chemical composition, and microbial communities

**DOI:** 10.3389/fmicb.2026.1925104

**Published:** 2026-08-31

**Authors:** Yinli Bi, Weixuan Guo, Li Xiao, Jianying Zhang, Yuguang Zhang, Dongdong Wang, Xintong Hu

**Affiliations:** 1 Institute of Ecological Environment Restoration in Mine Areas of West China, College of Geology and Environment, Xi’an University of Science and Technology, Xi’an, China; 2 State Key Laboratory for Fine Exploration and Intelligent Development of Coal Resources, China University of Mining and Technology, Beijing, China; 3 SDIC Hami Energy Development Co., Ltd., Xinjiang, China

**Keywords:** AMF, FTIR, leaf microbiota, litter decomposition, substrate quality

## Abstract

**Introduction:**

Arbuscular mycorrhizal fungi (AMF) are primarily recognized for their obligate symbiotic associations with the majority of terrestrial plants; however, an emerging body of research indicates their potential capacity to accelerate litter decomposition and facilitate the sequestration of soil organic carbon. Previous studies on AMF-mediated litter decomposition have primarily focused on the colonization of plant root systems, whereas mycorrhizal colonization of leaves has received comparatively limited attention. The effects of AMF leaf colonization on leaf substrate and microbial community structure remains largely understood due to limited direct evidence.

**Methods:**

In this study, a Petri dish experiment was conducted using in-situ soil, AMF inoculum and litter derived from *Amorpha fruticosa*, encompassing four treatments: CK (non-mycorrhizal substrate + surface-sterilized leaves), S (mycorrhizal substrate + surface-sterilized leaves), L (non-mycorrhizal substrate + non-surface-sterilized leaves), and SL (mycorrhizal substrate + non-surface-sterilized leaves).

**Results:**

With respect to litter decomposition and substrate stoichiometry, AMF specifically promoted the decomposition of aliphatic components in leaves, as reflected by a 12.75–24.58% reduction in the C–H/C=O ratio in AMF inoculation compared with AMF– treatments. Regarding microbial community shifts, AMF substantially elevated the abundance of *Hypocreales* (21.71- to 35.59-fold), *Sordariales* (2.79- to 12.57-fold) within the fungal community, and *Rhizobiales* (1.52- to 2.36-fold) within the bacterial community, facilitating the breakdown of recalcitrant carbon sources. In terms of carbon cycling implications, these findings suggest that AMF accelerated litter conversion into labile, high-quality material (lower C/N ratio), enhancing microbial utilization of aliphatic compounds and promoting saprophytic functional group proliferation, ultimately expediting litter decomposition.

**Discussion:**

Although this short-term experiment primarily captures the decomposition phase, the observed shifts in substrate chemistry and microbial processing provide a mechanistic basis for understanding how AMF may influence the quality and potential long-term stabilization of soil organic carbon.

## 1 Introduction

Climate warming constitutes one of the most urgent challenges facing humanity. In response to the severe threats posed by climate change and in alignment with the global temperature targets of 1.5 °C and 2 °C outlined in the Paris Agreement (Lee et al., 2023), achieving global carbon neutrality by the mid-century has emerged as a shared responsibility of the international community. Two fundamental strategies underpin this goal: emission reduction, which involves decreasing the release of carbon dioxide (CO_2_) into the atmosphere, and carbon sequestration, which enhances atmospheric CO_2_ absorption (Bian et al., 2022). At present, large-scale deployment of carbon capture, utilization, and storage technologies faces substantial technical and economic barriers, limiting their near-term potential as primary approaches for carbon sequestration. By contrast, terrestrial ecosystems offer a nature-based solution: leveraging plant–soil systems for carbon storage not only mitigates climate change but also represents one of the most promising pathways toward achieving carbon neutrality (Feng et al., 2024).

Soil organic carbon (SOC) constitutes the largest carbon reservoir within terrestrial ecosystems, storing over 2,300 Pg C (1 Pg = 1 × 10^15^ g), nearly four times the carbon content of plant biomass and three times that of the atmosphere (Zhang X. et al., 2022). The processes governing SOC formation and stabilization have attracted increasing attention in global change research. Approximately 70% of soil carbon flux originates from litter decomposition, with the litter layer representing the principal source of plant-derived carbon inputs (Bai et al., 2026). Litter exerts a critical influence on SOC stocks by regulating both the generation of new soil organic carbon and the mineralization of existing SOC. Even minor fluctuations in SOC stocks can substantially affect atmospheric CO_2_ concentrations (Zhang et al., 2014; Zhang X. et al., 2022). Accordingly, the decomposition of litter serves as a critical regulator of atmospheric CO_2_ dynamics (Cheng et al., 2026).

Litter decomposition is initially driven by physical and chemical processes, including the mechanical fragmentation of coarse litter into progressively finer particles and the concurrent dissolution and leaching of soluble constituents from the organic matrix (Giweta, 2020; Tan et al., 2021). Previous studies employing Fourier transform infrared (FTIR) spectroscopy have examined the effects of topographical slope and burial depth on litter decomposition rates, nutrient release patterns, and chemical structural evolution (Xue et al., 2019). Complementarily, researchers have applied ^13^C nuclear magnetic resonance spectroscopy to assess chemical composition changes during the degradation of wheat, soybean, and maize residues, demonstrating that O-alkyl carbon/alkyl carbon ratios and aromaticity critically influence the breakdown of macromolecular compounds (Xu et al., 2017). Accordingly, FTIR analysis of functional group relative contents provides a microscopic perspective on property variations during litter decomposition, which is also correlated with leaf stoichiometry.

Subsequently, biological processes and specifically microbial catabolism regulate the enzymatic breakdown and mineralization of the remaining complex and recalcitrant organic compounds by the soil microbiome (Giweta, 2020; Tan et al., 2021). At the local scale, litter decomposition rates are shaped by both the intrinsic characteristics of the litter mass and the specific microenvironmental conditions under which decomposition proceeds, with microbial communities functioning as the primary biological drivers orchestrating this process (Lang et al., 2021). Among these communities, soil fungi are considered the principal decomposers (Wang et al., 2015). By secreting an array of extracellular functional enzymes, these microorganisms decompose structurally complex organic polymers, including cellulose and lignin, thereby driving the transformation, oxidation, and reconfiguration of critical functional groups throughout the decomposition process.

Arbuscular mycorrhizal fungi (AMF) constitute a widespread group of soil-borne fungi that establish obligate symbiotic associations with the majority of dominant terrestrial plant species (Cheng et al., 2012; Brundrett and Tedersoo, 2018). Through the development of extensive extraradical hyphal networks and the release of bioactive exudates, AMF substantially enhance host plant stress resilience (Li et al., 2024) and facilitate the accumulation and stabilization of SOC within terrestrial ecosystems (Bi et al., 2025). Empirical observations further demonstrate that AMF display a pronounced preference for colonizing plant systems supplemented with litter inputs, implying that litter-derived carbon serves as a pivotal resource underpinning their growth, metabolic activity, and reproductive performance (Hodge et al., 2001). Although most investigations have traditionally centered on the colonization of plant root systems by AMF, accumulating evidence indicates that AMF are also capable of directly colonizing litter leaves within the topsoil layer adjacent to plant roots (Aristizábal et al., 2004). As obligate biotrophs lacking the enzymatic machinery to decompose organic matter, AMF are theoretically incapable of persisting in dead substrates; yet molecular and microscopic evidence confirms their rapid and widespread colonization of fallen leaves across diverse ecosystems (Bunn et al., 2019). This contradiction remains unresolved.

Previous studies indicate that AMF promote the decomposition of high-quality litter characterized by low C/N ratios (Jansa et al., 2019), while inhibiting the decomposition of low-quality litter with high C/N ratios. Nevertheless, some studies have revealed that the effect of AMF on litter decomposition depends not only on litter quality, but also on the decomposition stage; that is, AMF may stimulate decomposition during early stages and suppress it during later stages (Hodge and Fitter, 2010; Leifheit and Rillig, 2016). Concurrently, a meta-analysis conducted in 2024 demonstrated that AMF indeed promote decomposition more effectively under low substrate C/N ratios, but also noted that no significant difference exists between AMF and ectomycorrhizal (ECM) fungi regarding their effects on decomposition (Choreño-Parra and Treseder, 2024).

Under nutrient-deficient soil conditions, AMF can strategically adjust to resource scarcity by intensifying their colonization of litter substrates, thereby enhancing the acquisition efficiency of essential nutrients necessary to sustain their growth, metabolic performance, and symbiotic functioning (Hodge et al., 2001; Bunn et al., 2019). Meanwhile, a recent study suggested that AMF preferentially colonize high-quality litter to rapidly access nutrients rather than acting as saprotrophic decomposers, and that this phenomenon has been observed across different plant species and stages of decomposition (Lima et al., 2025). In Colombia, researchers also found typical structures of AMF in decomposing leaves, with litter colonization rates as high as 36.2–69.91%, and colonization was present from the early stages of decomposition through to the highly fragmented late stages (Díaz-Ariza et al., 2021). Upon colonizing leaf litter, AMF can directly alter the physical architecture of the substrate while concurrently releasing a diverse array of low-molecular-weight metabolites into the surrounding microenvironment. For instance, AMF release carboxylates to recruit bacteria recruit alkaline phosphatase–producing bacteria to concentrate around litter particles, while simultaneously suppressing microorganisms that are not functionally involved in decomposition (Zhang et al., 2018; Zhang L. et al., 2022). Such selective recruitment promotes the establishment of a functionally specialized microbial consortium, thereby enhancing the efficiency and coordination of litter breakdown processes (Bunn et al., 2019). In addition, the turnover and deposition of senescent AMF hyphae provide supplementary nutrient inputs to resident microbial communities, mitigating nutrient constraints and sustaining ongoing decomposition activity (Nuccio et al., 2013). Collectively, AMF-derived compounds stimulate basal microbial metabolic activity, reinforce the predominance of key bacterial decomposers, and ultimately exert regulatory control over the trajectory and intensity of litter decomposition dynamics.

Despite growing recognition that AMF colonize leaf litter and influence decomposition, previous studies have been predominantly conducted in natural field systems, where the direct effects of AMF are confounded by complex soil biotic and abiotic factors. Moreover, most existing work has focused on endpoint measurements of mass loss or nutrient release, without disentangling the mechanistic pathways linking AMF colonization, microbial community reassembly, and litter stoichiometric changes. We proposed to conduct controlled micro-experiments in Petri dishes. By simultaneously tracking AMF colonization dynamics, shifts in key saprotrophic microbial communities, and temporal changes in litter stoichiometry, this study represents a refined experimental dissection of AMF-mediated decomposition processes. Furthermore, by framing these mechanisms within the context of carbon turnover efficiency and carbon neutrality, our work offers novel insights into the microscale regulation of litter decomposition by AMF. We hypothesized that: (1) AMF were capable of colonizing litter, thereby altering litter stoichiometry (e.g., reducing the C/N ratio), which in turn accelerated litter decomposition; and (2) AMF colonization was associated with increased abundance of key saprophytic functional microbial communities, which may have contributed to litter decomposition and altered carbon distribution within the system.

## 2 Materials and methods

### 2.1 Materials

The soil used in the experiment was in-situ soil from the Daliuta coal mining subsidence area (39°14′–39°17′N, 110°10′–110°16′E), located in Shenmu County, Shaanxi Province, northwest China. The entire sample was autoclaved at 121 °C for 2 h, then air-dried and passed through a 2-mm sieve for subsequent use.

The strain employed in this study was *Funneliformis mosseae*, obtained from the Institute of Plant Nutrition, Resources and Environment, Beijing Academy of Agriculture and Forestry Sciences, and subsequently propagated under controlled conditions in our laboratory. The fungal strain was propagated for 60 days in our laboratory on host plants *Zea mays L.* and *Medicago sativa L.* Seeds were surface-sterilized with 10% H₂O₂ prior to sowing. The propagation substrate consisted of sand passed through a 2-mm sieve and autoclaved at 121 °C, amended with AMF inoculum at a ratio of 1 kg sand to 50 g inoculum (20:1, w/w). The AMF inoculum consisted of a mixture of spores, hyphal fragments, and mycorrhizal root segments. A portion of the AMF inoculum was sterilized at 121 °C for 2 h, air-dried, and sieved (2 mm) to prepare the non-mycorrhizal substrate. Sterilized in-situ soil (15 g) was thoroughly homogenized with 5 g of either mycorrhizal or non-mycorrhizal inoculum (3,1, w/w) to constitute the experimental substrate. The resulting spore density was determined to be 52 spores per gram. The basic physicochemical properties of substrate are presented in Supplementary Table S1. It should be noted that autoclaving may alter the chemical forms of carbon present in the substrate, and live inoculum may contain microorganisms other than *F. mosseae*. Thus, differences between live and autoclaved treatments reflect the net effect of the AMF inoculation, rather than the isolated effect of *F. mosseae* alone.

The experimental litter consisted of leaves from *Amorpha fruticosa*, collected from the Daliuta coal mining subsidence area, located in Shenmu County, Shaanxi Province, northwest China. Fifteen *Amorpha fruticosa* trees were randomly selected from the plantation, collected and pooled to minimize inter-plant variation in initial chemistry. The collected leaves were subsequently dried at 60 °C to constant weight and thoroughly homogenized prior to experimental setup to ensure uniformity of the litter material. As autoclaving (121 °C,20 min) can cause leaf browning and affect the structure of organic matter, in this study, a selection of leaves from litter was taken, soaked in 70% ethanol for 1 min, then soaked in a 10% H_2_O_2_ for 10 min (Setamam, 2024). The leaves were subsequently rinsed three times with sterile distilled water, dried, and set aside as surface-sterilized leaves for use. The effectiveness of the surface sterilization protocol was verified as follows. A 100 μL aliquot of the final rinse water was plated onto potato dextrose agar (PDA) plates, which were subsequently incubated at 28 °C for 72 h. No microbial growth was observed on any plate, confirming that the initial epiphytic microbial load had been effectively removed.

### 2.2 Experimental design

The experiment was conducted using Petri dishes (diameter: 9 cm), each containing 20 g of substrate (15 g sieved soil mixed thoroughly with 5 g mycorrhizal or non-mycorrhizal inoculum) evenly distributed across the base. A uniform layer of 0.3 g of litter was subsequently placed over the substrate. All Petri dishes were maintained in an artificial climate chamber at 25 °C in the absence of light, with leaf surfaces kept consistently moist throughout the experimental period. Four treatments were established: CK (non-mycorrhizal substrate + surface-sterilized leaves), S (mycorrhizal substrate + surface-sterilized leaves), L (non-mycorrhizal substrate + non-surface-sterilized leaves), and SL (mycorrhizal substrate + non-surface-sterilized leaves). Initial sampling from the microcosms was performed 30 days (T30) after incubation, with subsequent samplings conducted at 45 days (T45), 60 days (T60), 75 days (T75), and 90 days (T90). Each treatment was replicated three times, resulting in a total of 60 experimental units (4 treatments × 5 sampling times × 3 replicates).

Destructive sampling was employed, during which leaves and microbial agents were carefully separated and collected. The harvested litter was divided into two portions: one portion was dried at low temperature, weighed, and then entirely passed through a 0.15-mm sieve to determine basic physicochemical properties, while the other portion was stored at −80 °C for subsequent high-throughput sequencing. Similarly, the substrate was divided into two fractions: one was air-dried naturally for physicochemical analyses, and the remaining portion was stored at −80 °C for molecular studies.

### 2.3 Laboratory analyses

Spores were isolated using the wet sieving–decantation method combined with sucrose centrifugation (Gerdemann and Nicolson, 1963; Jenkins, 1964). Following sedimentation and stratification, the supernatant was collected and filtered. Spore counts were quantified under a stereomicroscope (SMZ-168, MOTIC China Group Co., Ltd.) (Han et al., 2019). Sections measuring 5 mm × 5 mm were excised from both sides of the main leaf vein of mechanically intact leaves. After fixation, dehydration, and drying, the samples were examined using a field emission scanning electron microscope (JSM-7610F, JEOL Ltd.) (Liu et al., 2020).

For assessment of AMF colonization, litter samples were immersed in 20% (w/v) KOH solution at 90 °C for 2 h. The clarified leaves were then acidified with 1% HCl for 1 min and stained in 0.05% trypan blue solution in lactic acid–glycerin for 6 h. After staining with trypan blue, samples were destained in a solution of distilled water, glycerol, and lactic acid (1:1:1, v/v/v) for 72 h. Samples were examined under a microscope (400×), and leaf colonization was quantified using the gridline intersect method (Phillips and Hayman, 1970).

Organic carbon content in the substrate (SOC) was determined using the Walkley–Black method. Total phosphorus in the substrate (STP) and in leaves (LTP) was measured by inductively coupled plasma optical emission spectrometry (ICP-OES, Optima 5,300 DV, PerkinElmer, Waltham, MA) (Bai et al., 2018). Total nitrogen in the substrate (STN) was quantified using the Modified Kjeldahl method (HJ 717–2014) (Zeng et al., 2019). Leaf total carbon (LTC) and leaf total nitrogen (LTN) were analyzed with an elemental analyzer (vario MACRO cube, Elementar).

For FTIR analysis, litter powder (1 mg, <200 mesh) was homogenized with 100 mg of dried KBr and pressed into a pellet. Spectra were recorded across the range of 4,000–400 cm^−1^ at a resolution of 4 cm^−1^ with 16 scans per sample. The relative peak area of functional group-associated bands were calculated as the ratio of individual peak areas to the total peak area.

Total genomic DNA samples were extracted using the MagBeads FastDNA Kit for Soil (116564384) (MP Biomedicals, CA, USA), following the manufacturer’s instructions. DNA was extracted from 0.5 g of fresh leaf tissue stored at −80 °C following the manufacturer’s instructions. The quality and concentration of the extracted DNA were subsequently assessed using a NanoDrop NC2000 spectrophotometer (Thermo Fisher Scientific, Waltham, MA, USA).

PCR amplification of the bacterial 16S rRNA gene V5–V7 region was performed using the forward primer 799F (5′-AACMGGATTAGATACCCKG-3′) and the reverse primer 1193R (5′-ACGTCATCCCCACCTTCC-3′). The fungal community was characterized by PCR amplification of the ITS1 region, employing the forward primer ITS1F (5′-CTTGGTCATTTAGAGGAAGTAA-3′) and the reverse primer ITS2R (5′-GCTGCGTTCTTCATCGATGC-3′). Briefly, PCR was carried out in a 25-μL mixture containing 0.25 μL of Q5 High-Fidelity DNA Polymerase, 5 μL of 5 × Reaction Buffer, 5 μL of 5 × High-Fidelity GC Buffer, 2 μL of 10 mM dNTPs, 1 μL of each 5 μM forward and reverse primer, 2 μL of DNA template, and 8.75 μL of ddH₂O. Thermal cycling consisted of initial denaturation at 98 °C for 5 min, followed by 25 cycles consisting of denaturation at 98 °C for 30 s, annealing at 53 °C for 30 s, and extension at 72 °C for 45 s, with a final extension of 5 min at 72 °C. PCR amplicons were purified with Vazyme VAHTSTM DNA Clean Beads (Vazyme, Nanjing, China) and quantified using the Quant-iT PicoGreen dsDNA Assay Kit (Invitrogen, Carlsbad, CA, USA). After the individual quantification step, amplicons were pooled in equal amounts, and pair-end 2250 bp sequencing was performed using the Illlumina NovaSeq platform with NovaSeq 6000 SP Reagent Kit (500 cycles) at Shanghai Personal Biotechnology Co., Ltd. (Shanghai, China). The raw sequences were submitted to the NCBI database under the BioProject accession number PRJNA1503789.

### 2.4 Statistical analysis

All results are reported as mean ± standard deviation (SD) of replicate measurements. Normality and homogeneity of variance were assessed using the Shapiro–Wilk and Levene tests, respectively. Variables that deviated from normality were log-transformed prior to analysis. The effects of different treatments on basic physicochemical properties and microbial communities were assessed using one-way analysis of variance (ANOVA) and Student’s *t*-test. Pairwise comparisons among the effects of AMF inoculation, leaf surface sterilization, and sampling time were conducted using least significant difference (LSD) *post-hoc* test at a significance threshold of *p* < 0.05. All statistical analyses were performed using IBM SPSS Statistics 26. FTIR spectral analyses were carried out in Origin 2021, with baseline correction applied to all absorption peaks prior to calculation of individual peak areas.

Raw sequencing data were processed using QIIME2 (version 2024.5). Adapter sequences and low-quality reads were removed, and denoising, merging, and chimera filtering were performed using the DADA2 plugin to obtain high-quality amplicon sequence variants (ASVs) (Callahan et al., 2016). Taxonomic assignment of bacterial and fungal sequences was performed using the SILVA 132 and UNITE 8.0 databases, respectively (Kõljalg et al., 2013). Alpha diversity indices (e.g., Chao1, Shannon) were calculated using QIIME2. Beta diversity was evaluated based on the Bray–Curtis distance matrix using the vegan package in R (version 4.3.3), visualized through principal coordinates analysis (PCoA), and statistically tested via permutational multivariate analysis of variance (PERMANOVA) using the adonis2 function with 999 permutations. LEfSe analysis was performed using the Python LEfSe package in conjunction with R (version 4.3.3). Mantel tests and redundancy analysis (RDA) were conducted in R (version 4.5.2).

## 3 Results

### 3.1 Litter decomposition

As decomposition progressed, litter mass decreased progressively across all treatments. The overall order of litter mass loss was as follows: S > SL > L > CK. The decomposition rate exhibited an initial increase, followed by a gradual plateau over time (Supplementary Figure S3).

Observation of leaf surfaces via scanning electron microscopy (SEM) revealed that AMF colonized in an outward-to-inward pattern. The AMF-like hyphae observed were relatively thick and exhibited morphological characteristics resembling those of runner hyphae (RH). Distinct differences were observed in the distribution patterns of RH and other microorganisms on leaf surfaces across different time points (T30, T45, T60, T75, T90) and treatments (SL, S, L, CK).

**Figure 1:**
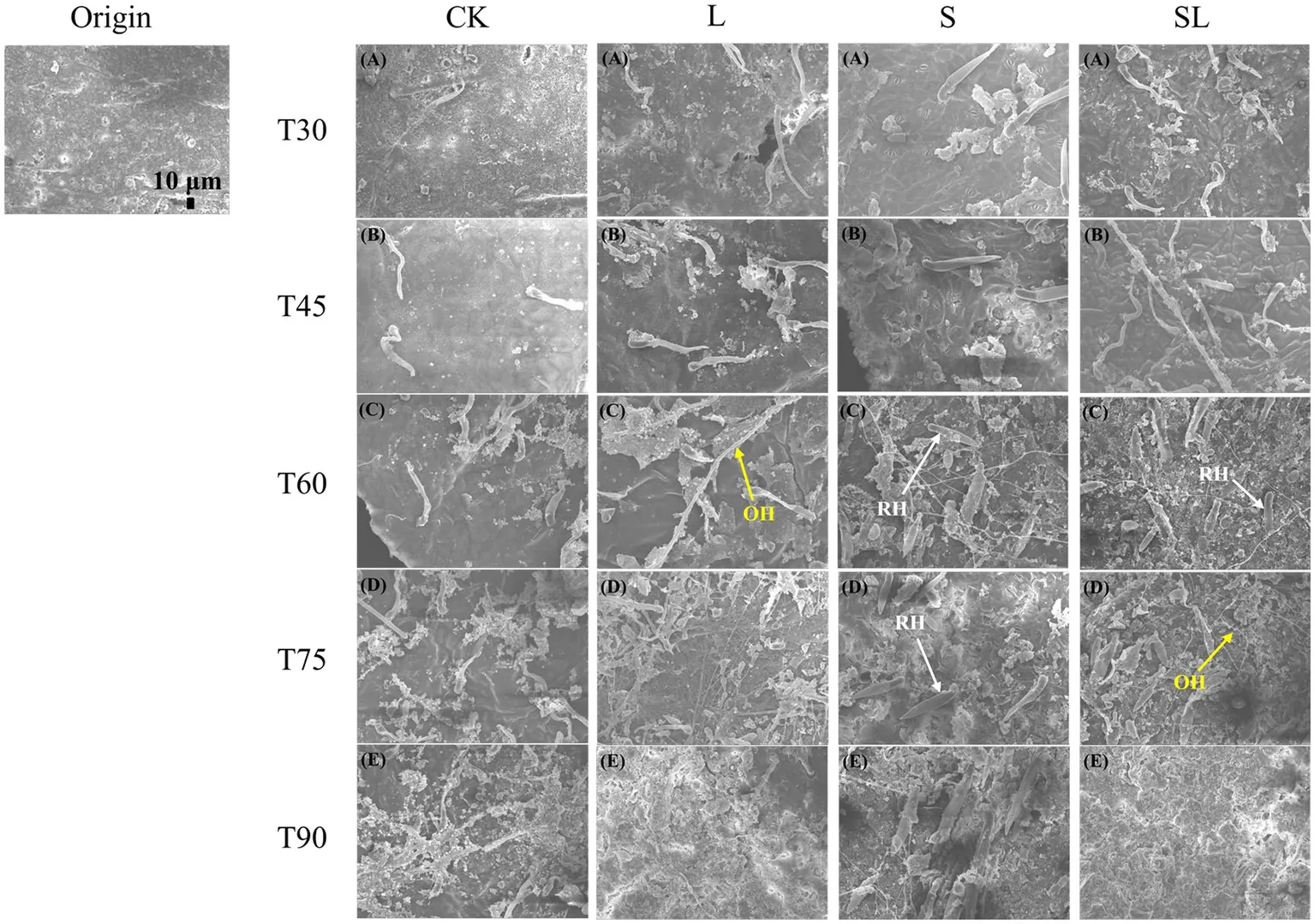
SEM images of different treatments. All images at the same magnification (×500); scale bar = 10 μm. RH, Runner hyphae; OH, Other fungal hyphae. **(A)** 30 days; **(B)** 45 days; **(C)** 60 days; **(D)** 75 days; **(E)** 90 days.

At T30, the L treatment exhibiting the largest growth area of other microorganisms among all treatments. During T45 and T60, RH, other microbial hyphae, and their respective coverage areas all increased synchronously in the S and SL treatments. By T75, RH on the leaf surfaces of the S treatment remained plump and well-developed; in contrast, the number of AMF hyphae in SL treatments decreased, and the hyphae appeared shriveled. Concurrently, the abundance and coverage of other microbial hyphae increased. At T90, RH on leaf surfaces in the S treatment also displayed a shriveled state (Figure 1).

### 3.2 Substrate nutrients and leaf stoichiometry

As decomposition progressed, SOC content across all treatments initially declined and subsequently increased. The lowest SOC value was recorded in the S treatment at T30, whereas the highest value was observed in the SL treatment at T90. STN and STP in the substrate exhibited a similar trend, with the minimum value occurring in the S treatment at T30 and the maximum in the S treatment at T90.

During the initial 30-day period, the C/N ratio of litter decreased rapidly across all treatments. Notably, leaf C/N ratios in the S and SL treatments remained consistently lower than those in the CK and L treatments, maintaining values around 9–10 (Supplementary Figure S4).

### 3.3 Changes in functional groups during litter decomposition

FTIR spectra of litter at different stages of decomposition were analyzed to identify the primary chemical constituents. The intense and broad absorption band near 3,375 cm^−1^ was attributed to O–H stretching vibrations. Absorption bands in the range of 2,800–3,000 cm^−1^ corresponded to symmetric and asymmetric C–H stretching vibrations. The band around 1,660 cm^−1^ was associated with C=O stretching vibrations, while the band at approximately 1,525 cm^−1^ corresponded to C=C stretching vibrations. Finally, the absorption band near 1,040 cm^−1^ was linked to C–O stretching vibrations (Supplementary Table S2; Supplementary Figure S5).

The relative peak area of functional group-associated bands in litter followed the order: O–H > C–O > C=O > C=C > C–H during the decomposition process (Supplementary Figure S6). Analysis of significant differences among the five absorption peak areas indicated that the O–H peak differed significantly from all other relative peak area of functional group-associated bands (*p* < 0.05). In addition, the C–O peak showed significant differences when compared with the C=C and C–H peaks (*p* < 0.05).

**Figure 2:**
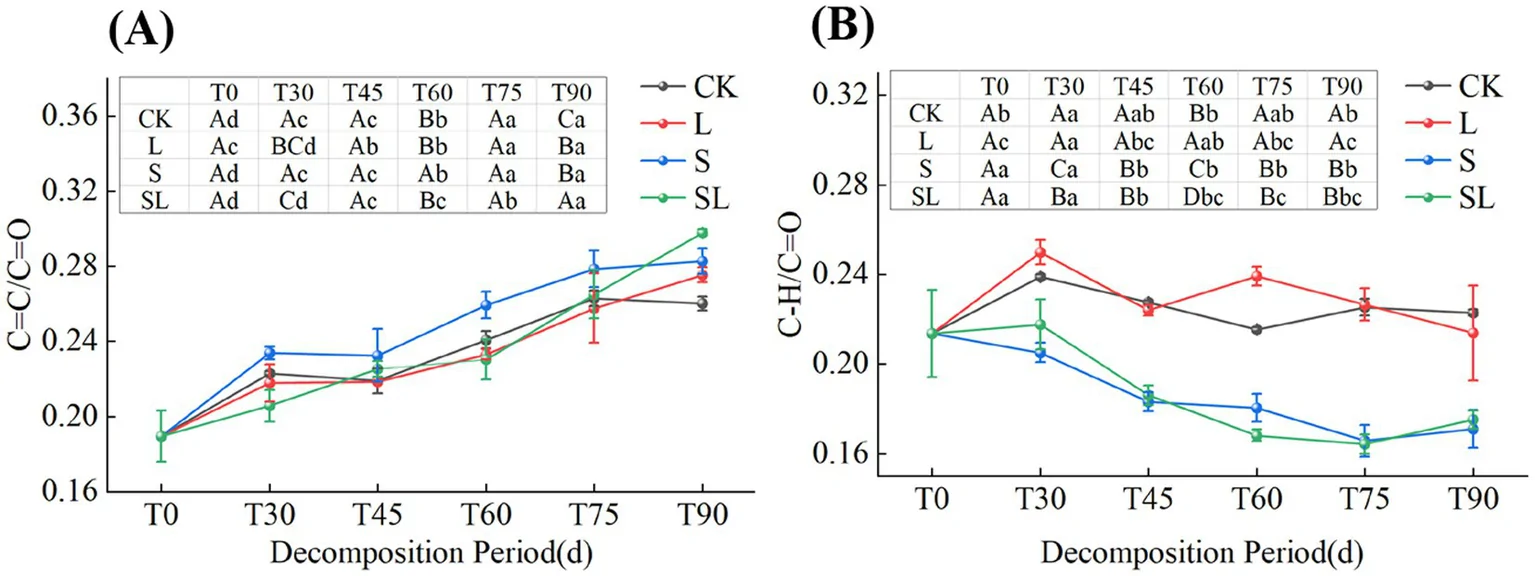
Differences in FTIR functional group ratios under different treatments. **(A)** C=C/C=O; **(B)** C-H/C=O. CK: non-mycorrhizal + surface-sterilized leaves, S: mycorrhizal + surface-sterilized leaves, L: non-mycorrhizal + non-surface-sterilized leaves, and SL: mycorrhizal + non-surface-sterilized leaves. Mean (n=9) values ± SE are shown. Values with different letters are significantly different at *p* < 0.05 (Uppercase letters: different treatments at the same time, lowercase letters: different time points in the same treatment, shared letters indicate no significant difference). Subsequent figures follow the same method.

During the decomposition process, the C=C/C=O ratio consistently increased across all treatments, with the S treatment exhibiting higher values than the other treatments, except at T90. By the end of decomposition, the C=C/C=O ratio followed the order SL > S > L > CK, suggestive of a relative enrichment in aromatic moieties compared to carbonyl-containing compounds, particularly in the S and SL treatments. Concurrently, the C–H/C=O ratio decreased in all treatments as decomposition progressed. The sterilized AMF treatments (CK and L) consistently displayed higher C–H/C=O ratios than the AMF inoculation treatments (S and SL) throughout the decomposition period. After AMF inoculation, this ratio declined by 12.75 to 24.58%, consistent with a relative decrease in aliphatic structures or an increase in oxygenated functional groups. These results observed by FTIR suggests that AMF inoculation may have promoted the decomposition of aliphatic components, thereby resulting in a relative increase in the proportion of aromatic constituents in the leaf litter (Figure 2).

### 3.4 Microbial changes during litter decomposition

**Figure 3:**
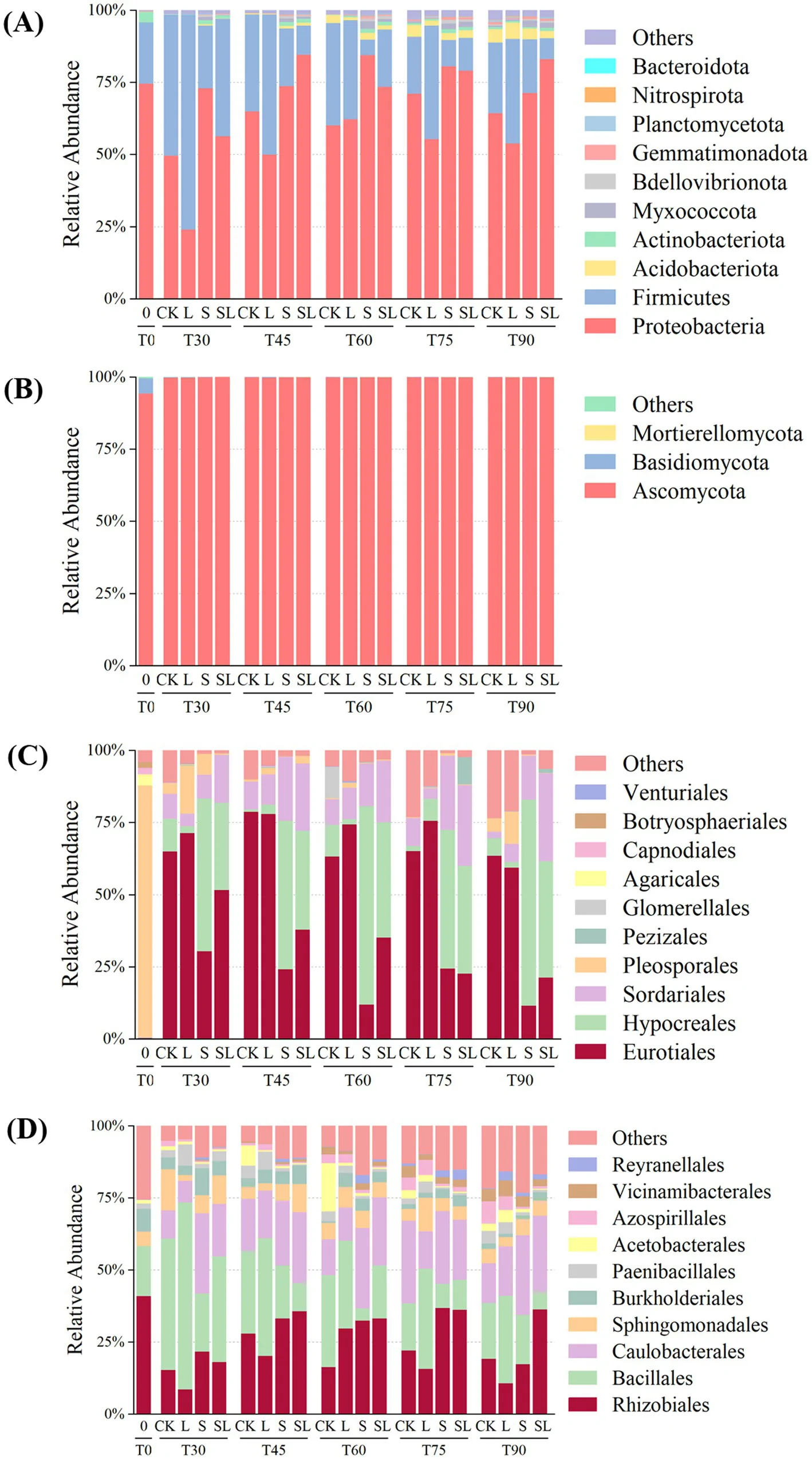
Microbial species composition in litter under different treatments. **(A)** Bacteria—phylum level; **(B)** Fungi—phylum level; **(C)** Bacteria—order level; **(D)** Fungi—order level.

High-throughput sequencing of bacterial communities across the various treatments identified a total of 18 phyla, 45 classes, 113 orders, 190 families, 361 genera, and 602 species. In contrast, fungal high-throughput sequencing revealed six phyla, 18 classes, 49 orders, 101 families, 203 genera, and 310 species across the different treatments. At the phylum level, *Ascomycota* was the overwhelmingly dominant fungal group, representing more than 99% of the average relative abundance, followed sequentially by *Basidiomycota*, *Mortierellomycota*, and other unclassified taxa. For bacteria, *Proteobacteria* and *Firmicutes* were the most abundant phyla, collectively accounting for more than 90% of the total relative abundance, followed by *Acidobacteria*, *Actinomycota*, and other groups. Over the course of decomposition, the relative abundance of *Acidobacteria* consistently increased, with the most pronounced growth observed in the L treatment, whereas fungal phyla exhibited a gradual decline as decomposition progressed (Figures 3A,B).

At the order level, *Eurotiales* and *Hypocreales* were the predominant fungal orders, collectively comprising over 70% of the average relative abundance, followed by *Sordariales*, *Pleosporales*, and additional minor groups. The relative abundance of *Eurotiales* was significantly lower in the S and SL treatments compared to the CK and L treatments, whereas *Hypocreales* and *Sordariales* showed significantly higher abundances in the S and SL treatments relative to the CK and L treatments. During the decomposition process, the relative abundances of *Hypocreales* and *Sordariales* continued to increase within the S and SL treatments. At T90, the S treatment exhibited the highest relative abundance of *Hypocreales* at 71.3%, whereas the SL treatment displayed the highest relative abundance of *Sordariales* at 30.4%. Inoculation with AMF led to a 21.71- to 35.59-fold increase in *Hypocreales* abundance and a 2.79- to 12.57-fold increase in *Sordariales* abundance.

Among bacteria, the most prevalent orders were *Rhizobiales*, *Bacillales*, and *Caulobacterales*, together contributing more than 68% of the total relative abundance, with *Sphingomonadales*, *Burkholderiales*, and other orders making up the remainder. Throughout all time points, the relative abundance of *Rhizobiales* in the S and SL treatments was consistently and significantly higher than in the CK and L treatments, with post-inoculation increases ranging from 1.52- to 2.36-fold (Figures 3C,D). Conversely, *Bacillales* showed markedly higher relative abundance in the CK and L treatments compared to the S and SL treatments. Notably, the *Caulobacterales* order, which was absent in the initial leaf samples, emerged in all treatments as decomposition progressed, indicating its gradual colonization and potential role in the decomposition process.

### 3.5 Prediction of leaf microbial function

**Figure 4:**
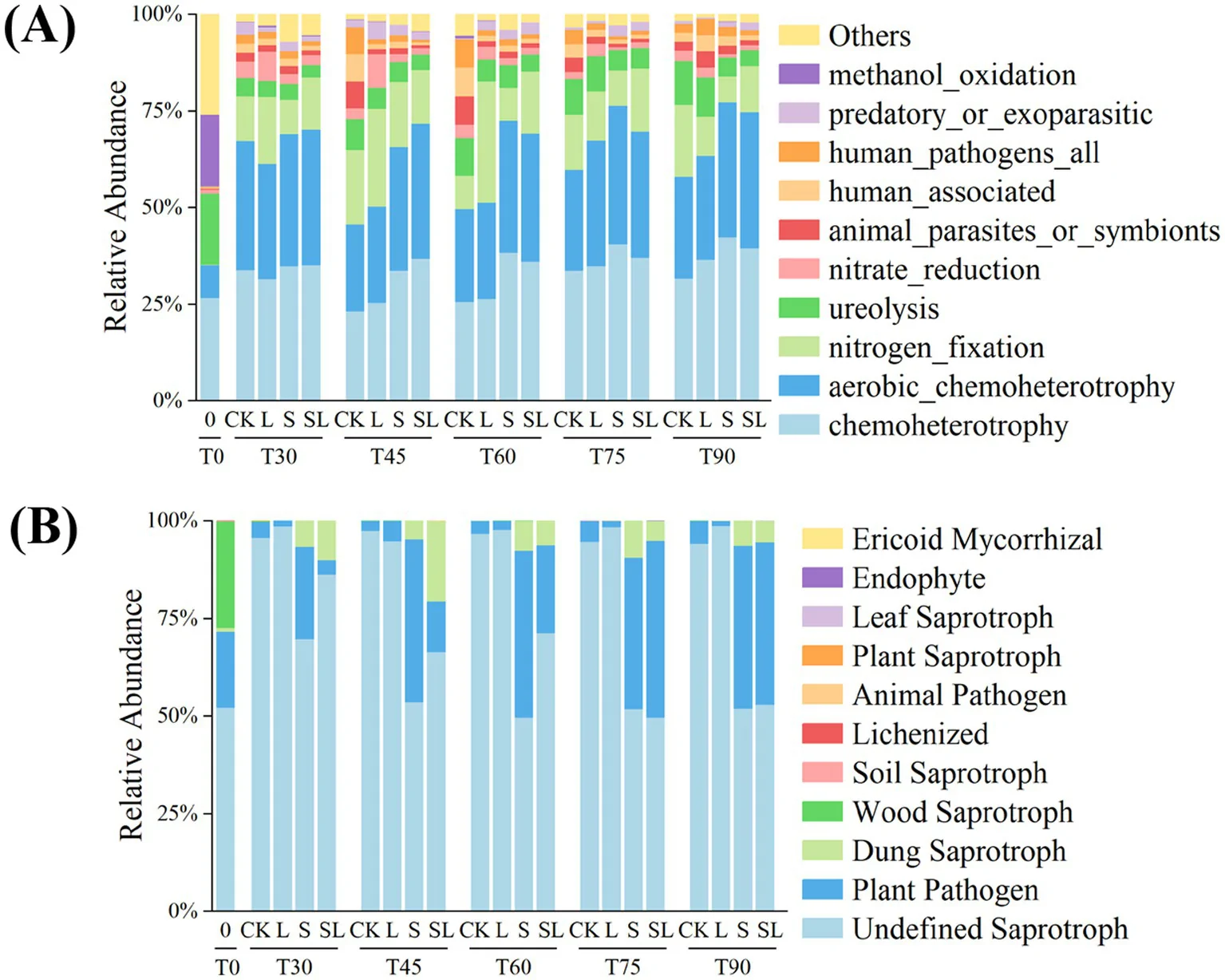
Functional prediction of **(A)** bacterial and **(B)** fungal communities in litter of different treatments.

Functional prediction and annotation of litter bacterial communities were conducted using FAPROTAX, with the top 10 functional groups selected based on their relative abundance to serve as the primary functional categories. The results revealed that the predominant functions across all treatments were chemoheterotrophy and aerobic chemoheterotrophy, which together accounted for 45.6 to 77.3% of the total relative abundance. As decomposition progressed, the S and SL treatments showed an increase in the proportion of these two functions. In addition, decomposition enhanced the relative contributions of nitrate reduction and nitrogen fixation, while concurrently reducing the proportion of ureolysis (Figure 4A).

Based on FUNGuild predictions of the ecological functions of fungal communities in leaves under various treatments, the primary ecological functions identified were undefined saprotroph (a general saprobe, or in cases where the ecology is not known but suspected to be a saprobe), plant pathogen, and dung saprotroph. In contrast, other functional groups displayed average relative abundances below 1%. The relative abundance of taxa assigned to the plant pathogen and dung saprotroph guilds increased during decomposition, particularly in the S and SL treatments.

Conversely, all treatments led to a decrease in the relative abundance of wood saprotrophs (Figure 4B).

### 3.6 Correlation between microbial community structure and physicochemical factors

**Figure 5:**
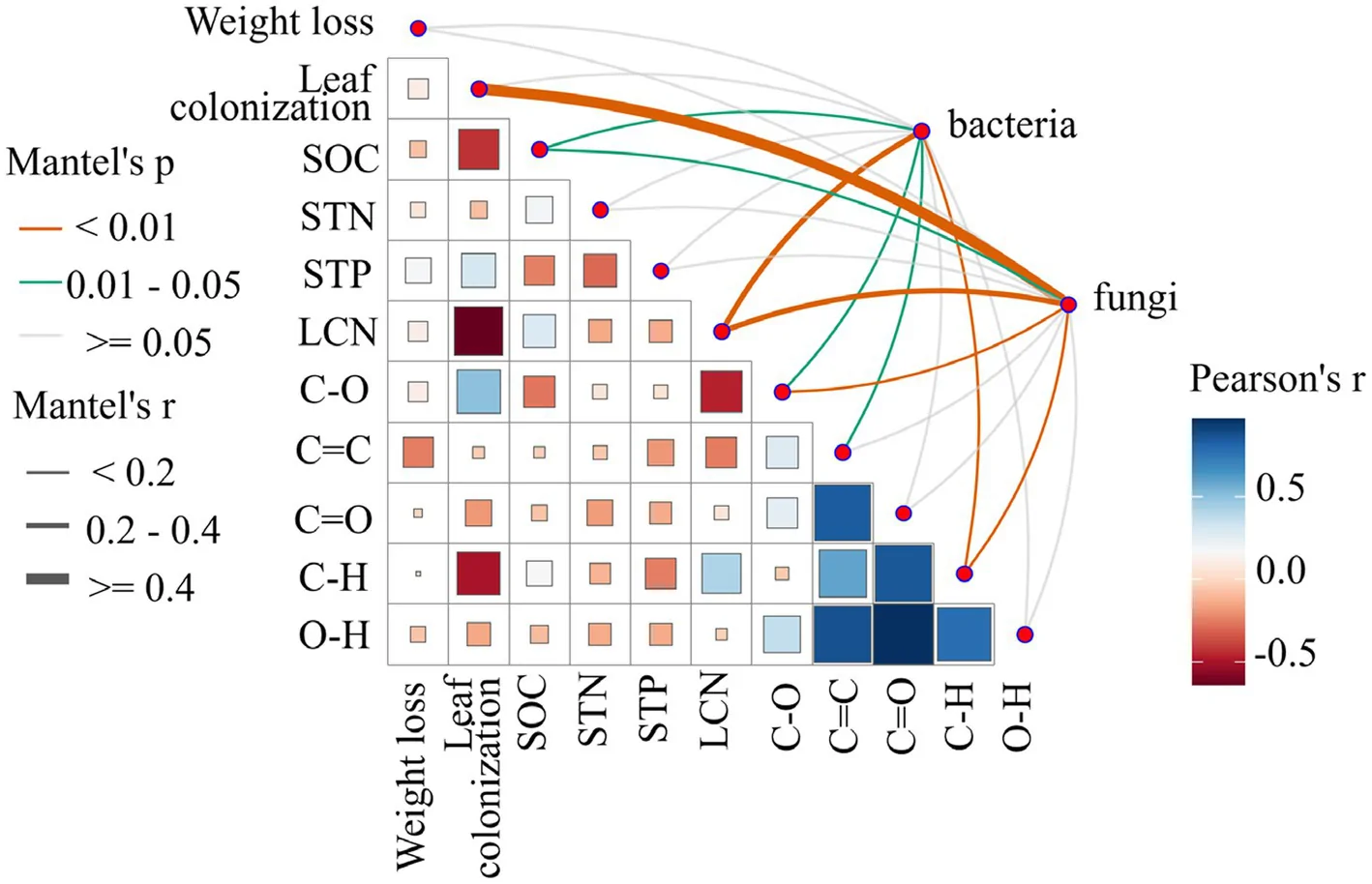
Mantel tests analysis of microbial communities and physicochemical factors under different treatments.

The Mantel test revealed correlations between litter bacterial and fungal communities and key physicochemical factors. Bacterial communities exhibited a highly significant and moderate positive correlation with the litter C/N ratio (*r* = 0.34, *p* = 0.001), as well as a similarly significant positive correlation with C–H content (*r* = 0.16, *p* = 0.008). In addition, bacterial communities displayed a significant positive correlation with C–O content (*r* = 0.13, *p* = 0.008) and a notable positive correlation with litter C=C content (*r* = 0.10, *p* = 0.042). In contrast, fungal communities showed a highly significant and relatively strong positive correlation with AMF colonization rate (*r* = 0.59, *p* = 0.001). They also exhibited a highly significant and moderate positive correlation with the litter C/N ratio (*r* = 0.36, *p* = 0.001) and a significant positive correlation with C–O content (*r* = 0.18, *p* = 0.001) (Figure 5).

## 4 Discussion

### 4.1 The effect of AMF on leaf stoichiometry and functional groups during the decomposition process

In our study, the treatment combining AMF with non-surface-sterilized leaves exhibited a lower leaf colonization rate compared to the AMF-only treatment (Supplementary Figure S2). This reduction was likely attributable to competition for ecological niches between AMF and leaf-associated saprophytic fungi. Previous studies have demonstrated that AMF share greater niche overlap with saprophytic fungi than with other microbial groups, which can suppress fungal activity and delay saprophyte development by approximately 1 month (Gui et al., 2020).

Fungal mycelia function as exploratory networks, capable of extending hyphae to locate and colonize new substrates (Vieira et al., 2025). Among these, RH are a specialized form of thick, aseptate vegetative hyphae, with high mechanical strength and elongation capacity. These features allow RH to penetrate host tissues or litter via structures such as plant vascular systems, serving as the primary hyphal type that expands colonization space during the early stages of AMF establishment. RH were distinguished from other fungal hyphae based on the following morphological criteria adapted: (1) relatively large (7–15 μm) and uniform diameter and (2) few or no branches. These features allowed differentiation from septate saprotrophic hyphae and from thinner AMF germ tubes (2–5 μm) or absorptive hyphal networks (2–4 μm). Our observations indicated that hyphae colonizing litter resembled RH in morphology but displayed fewer branches, likely reflecting the functional emphasis of RH on forming hyphal connections between hosts to enhance mycorrhizal network continuity. Additionally, the substantially larger diameter of RH compared with germ tubes imposed structural constraints that limit extensive branching (Friese and Allen, 1991). It should be noted that AMF identification in this study relied primarily on morphological characteristics observed in stained leaves (Supplementary Figure S7). Future studies employing AMF-specific markers are needed to further confirm AMF colonization and to elucidate their actual roles during the litter colonization process.

In the short term, the presence of AMF may induce a transient decline in SOC. However, over extended periods, mycorrhizal activity enhances the breakdown of both fresh litter and topsoil organic matter, ultimately promoting the accumulation and long-term storage of SOC (Verbruggen et al., 2013). AMF are capable of directly assimilating inorganic nitrogen in the form of ammonium (NH₄^+^) and nitrate (NO_3_^−^), as well as small organic nitrogen molecules such as glycine and chitin. Moreover, through the release of hyphal exudates, AMF stimulate the proliferation of specific microbial communities, thereby influencing nutrient cycling and microbial dynamics (Wei et al., 2016). Observed trends in substrate nutrient content (Table 1) suggested that AMF-inoculated treatments preferentially utilized available nutrients within the substrate, facilitating both rapid AMF colonization and the expansion of other microbial populations. A possible synergistic interaction between AMF and other microbial populations could have promoted leaf litter decomposition, which might explain the observed increases in SOC, STN, and STP. We acknowledge that autoclaving may have altered nutrient availability in the control. Accordingly, our findings reflect the overall effect of AMF inoculation rather than the isolated impact of *F. mosseae*. Concurrently, the leaf C/N and C/P ratios progressively declined throughout the decomposition process (Supplementary Figure S4), reflecting the preferential consumption of labile carbon compounds, a process that was further accelerated by AMF presence. In short, AMF may influence decomposition indirectly through interactions with saprotrophic microorganisms and changes in nutrient.

**Table 1:** Substrate nutrients at different time points.

Time	SOC (g/kg)				STN (g/kg)				STP (g/kg)			
	CK	L	S	SL	CK	L	S	SL	CK	L	S	SL
T0	11.27 ± 2.18Aab	11.27 ± 2.18Ab	11.27 ± 2.18Ab	11.27 ± 2.18Ab	0.24 ± 0.03Aab	0.24 ± 0.03Aab	0.24 ± 0.03Aabc	0.24 ± 0.03Aab	0.24 ± 0.00Ad	0.24 ± 0.00Ac	0.24 ± 0.00Ae	0.24 ± 0.00Ad
T30	9.78 ± 0.90Bb	9.55 ± 0.70Bb	6.49 ± 1.30Ac	7.09 ± 0.96Ac	0.20 ± 0.00Ab	0.20 ± 0.02Ad	0.20 ± 0.01Ac	0.20 ± 0.02Ab	0.24 ± 0.00Ad	0.23 ± 0.01Ad	0.22 ± 0.01Bf	0.22 ± 0.00Be
T45	9.88 ± 0.39Ab	9.98 ± 0.80Ab	10.71 ± 0.49Ab	10.40 ± 0.69Ab	0.21 ± 0.01Ab	0.21 ± 0.01Acd	0.22 ± 0.03Abc	0.21 ± 0.05Aab	0.24 ± 0.00 Bd	0.25 ± 0.01Abc	0.26 ± 0.00Ad	0.26 ± 0.01Acd
T60	11.07 ± 1.45Aab	11.17 ± 2.60Ab	11.61 ± 0.68Ab	11.58 ± 1.44Ab	0.22 ± 0.00Ab	0.22 ± 0.01Abcd	0.23 ± 0.02Aabc	0.23 ± 0.04Aab	0.26 ± 0.00Cc	0.26 ± 0.00Bb	0.27 ± 0.00Ac	0.26 ± 0.00Bbc
T75	11.82 ± 0.40Aab	12.34 ± 1.00Aab	12.85 ± 1.76Aab	12.72 ± 0.37Aab	0.24 ± 0.03Aab	0.24 ± 0.01Aabc	0.25 ± 0.01Aab	0.25 ± 0.00Aab	0.27 ± 0.00Ab	0.27 ± 0.01Aab	0.28 ± 0.00Ab	0.27 ± 0.01Aab
T90	13.04 ± 0.48Aa	14.59 ± 1.50Aa	14.72 ± 2.51Aa	15.05 ± 1.58Aa	0.26 ± 0.02Aa	0.26 ± 0.00Aa	0.27 ± 0.03Aa	0.27 ± 0.00Aa	0.28 ± 0.00Ba	0.28 ± 0.00Ba	0.29 ± 0.00Aa	0.28 ± 0.00Ba

SOC, organic carbon content in the substrate; STN, total nitrogen in the substrate; STP, total phosphorus in the substrate; CK: non-mycorrhizal substrate + surface-sterilized leaves, S: mycorrhizal substrate + surface-sterilized leaves, L: non-mycorrhizal substrate + non-surface-sterilized leaves, and SL: mycorrhizal substrate + non-surface-sterilized leaves. T0, T30, T45, T60, T75, T90: At 0, 30, 45, 60, 75, and 90 days after the start of the experiment. Mean (*n* = 9) values ± SE are shown. Values with different letters are significantly different at *p* < 0.05 (Uppercase letters: different treatments at the same time, lowercase letters: different time points in the same treatment, shared letters indicate no significant difference).

Leaf litter consists of both structural constituents (primarily lignin, cellulose, and hemicellulose) and non-structural constituents (predominantly dissolved organic carbon, proteins, and lipids). Microbial communities readily assimilate small-molecule organic carbon compounds; however, macromolecular compounds require enzymatic hydrolysis into simpler molecules before they can be utilized (Cotrufo et al., 2015). During the first 30 days of decomposition, the process proceeded relatively rapidly, largely driven by the leaching of non-structural compounds under abiotic influences. From days 30 to 90, decomposition rates slowed as structural components became the primary substrates, with microbial activity playing a dominant role in their gradual breakdown (Supplementary Figure S3). While FTIR spectroscopy provides valuable information on the relative peak area of functional group-associated bands during litter decomposition, several limitations constrain its interpretive power. It merely serves as qualitative chemical evidence complementary to direct measurements of mass loss, stoichiometric changes, and microbial.

In this study, the C=C functional groups originated from the aromatic constituents of plant-derived organic matter (Zheng et al., 2021; Kablanbekov et al., 2023) and served as reliable chemical indicators of the persistence and stability of plant residues within soil environments. Microbially mediated degradation processes result in an increased relative peak area of C=O bonds (Maleke et al., 2019), reflecting the enzymatic oxidation of complex aromatic structures by microorganisms, which convert aromatic ring systems into simpler organic compounds containing carboxyl functional groups. Consequently, the C=O bond acts as a distinctive spectral marker indicative of microbial transformation. The ratio of C=C to C=O (C=C/C=O) provides a quantitative measure of the relative prevalence of intact plant-derived aromatic compounds compared to products generated through microbial processing, with higher ratios signifying a greater retention of original aromatic structures. Conversely, C–H bonds derive from aliphatic components within plant-derived organic matter, and the C–H/C=O ratio represents the balance between plant-derived aliphatic substances and microbial transformation products, with elevated ratios indicating a larger fraction of unaltered aliphatic compounds (Yu et al., 2022). Previous studies have documented that decomposition enhances the condensation and stability of aromatic compounds while reducing the abundance of lipids and waxes, reflecting the preferential microbial degradation of more labile constituents (Turrión et al., 2025). Aliphatic C–H bonds are more susceptible to biodegradation, and extensive litter decomposition facilitates the accumulation of aromatic compounds in deeper soil layers (Thai et al., 2024).

In accordance with these mechanisms, our results showed a progressive increase in the C=C/C=O ratio alongside a concomitant decrease in the C–H/C=O ratio throughout the litter decomposition process (Figure 2). This pattern likely reflected the preferential microbial degradation of aliphatic components, which simultaneously elevated the relative proportion of aromatic compounds, corroborating trends reported in previous studies.

### 4.2 The effect of AMF on microbial communities during decomposition processes

Microbial community structures displayed pronounced differences and distinct segregation among the treatments. Fungi have long been regarded as the primary drivers of litter decomposition (Purahong et al., 2014); however, the role of bacteria in this process should not be overlooked, as they contribute substantially to the breakdown of organic substrates (Gui et al., 2017). In the present study, litter decomposition was associated with a marked decline in fungal community richness, while bacterial community richness and diversity exhibited a steady and progressive increase over the course of decomposition (Supplementary Figure S8). As decomposition progressed, the relative abundance of *Ascomycota* within the fungal community reached a peak of 99% (Figure 3B), demonstrating that *Ascomycota* were the predominant fungal decomposers during the early stages of litter degradation (Unterseher et al., 2013). *Ascomycota* function as predominant saprotrophic fungi capable of decomposing leaf litter and releasing nutrients (Zhang et al., 2025). These nutrients can be utilized by microorganisms lacking saprotrophic abilities, including AMF. This may explain why AMF tend to colonize leaf litter at the initial stages of decomposition; however, such colonization tendency is strongly influenced by the litter C/N and C/P ratios, as these stoichiometric characteristics determine nutrient availability (Lima et al., 2025). Notably, previous studies have reported that inoculation with AMF can enhance the relative abundance of *Basidiomycota* (Gui et al., 2020), a pattern that contrasts with our observations. This discrepancy might be attributed to differences in litter quantity, substrate composition, and the temporal dynamics of decomposition. Functionally, *Ascomycota* primarily target labile substrates such as cellulose and hemicellulose, whereas *Basidiomycota* specialize in degrading more recalcitrant components, including lignin and humus, during the later stages of decomposition (Purahong et al., 2016). The absence of *Glomeromycota* in ITS sequencing data likely reflects the known bias of universal ITS primers against AMF rather than their true absence (Bellemain et al., 2010; Sene et al., 2025).

Among bacterial phyla, *Proteobacteria* and *Firmicutes* collectively accounted for more than 90% of the average relative abundance across all treatments (Figure 3A). During litter decomposition, *Proteobacteria* rapidly proliferate by utilizing readily soluble organic compounds (Guan et al., 2023) and concurrently secrete enzymes such as pectinase and hemicellulase, facilitating the disruption of plant cell walls (Singh et al., 2022; Zhang X. P. et al., 2022). In parallel, *Firmicutes* efficiently degrade cellulose and hemicellulose, thereby accelerating the overall decomposition of leaf litter (Chen et al., 2023). At the order level, the dominant bacterial groups included *Rhizobiales*, *Bacillales*, and *Caulobacterales* (Figure 3C). Members of the *Bacillales* order play a central role in decomposition by secreting extracellular enzymes that break down complex organic matter (Huang et al., 2023). Notably, the relative abundance of *Rhizobiales* was significantly higher in both the S and SL treatments compared to the CK and L treatments. *Rhizobiales* exhibit the functional capacity to convert organic nitrogen into forms that are readily accessible to other decomposing microorganisms, thereby enhancing soil nitrogen availability. This functional trait provides critical nutrient support for decomposition and promotes the efficient progression of auxiliary decomposition processes (Paulitsch et al., 2021; Xu et al., 2025). Furthermore, the nitrogen-fixing activity of *Rhizobiales* could partially explain the observed reduction in the litter C/N ratio, though this was not directly evaluated in the present study. Evidence suggests that AMF interact with the soil bacterial community to regulate nitrogen cycling during litter decomposition, which may alter the local availability of nitrogen resources (Nuccio et al., 2013; Purahong et al., 2016).

Interestingly, decomposition resulted in an increase in the relative abundance of the bacterial order Caulobacterales (Parthiban and Buvaneswari, 2025). This order plays a regulatory role in maintaining environmental pH and redox potential, thereby creating favorable conditions for decomposer microorganisms. Within the fungal community, *Hypocreales* and *Sordariales* were identified as the two dominant orders (Figure 3D). Both groups possess the functional capacity to secrete comprehensive degradative enzyme systems, catalyzing the breakdown of complex organic compounds in litter and promoting the transformation of litter into humus (Berlemont et al., 2014; Strakowska et al., 2014). Notably, AMF inoculation markedly increased the relative abundance of these two functional fungal groups, thereby providing critical support for the efficient decomposition of late-stage, recalcitrant litter components.

Changes in microbial community structure generally act as the primary drivers of shifts in ecological function (Lilja and Johnson, 2016). Therefore, establishing a clear linkage between community structure and ecological function is essential to elucidate the intrinsic mechanisms by which AMF facilitate litter decomposition through modulation of leaf-associated microbial communities. Functional predictions for bacterial communities (Figure 4A) revealed that AMF significantly enhanced the relative proportions of two dominant metabolic functions: chemoheterotrophy and aerobic chemoheterotrophy. Chemoheterotrophy represents a central metabolic pathway that enables microorganisms to efficiently utilize organic carbon substrates (Yan et al., 2025). The observed increases in the functional contributions of nitrate reduction and nitrogen fixation likely contributed to enhanced nitrogen content in leaves, consistent with the trends observed in leaf C/N ratios.

Similarly, functional predictions for fungal communities indicated that AMF increased the relative abundance of taxa assigned to the plant pathogen and dung saprotroph guilds (Figure 4B). It reflectd the concomitant rise in the relative abundance of the fungal order Sclerotiorales under AMF inoculation. Litter decomposition promotes bacterial groups associated with chemoheterotrophy and aerobic chemoheterotrophy. It also enhances fungal groups such as pathogens and saprotrophs (Zhang et al., 2023). These patterns corroborate our observations.

Prior research has highlighted the substantial influence of fungal communities on AMF colonization (Gui et al., 2017; Gui et al., 2020). Consistently, Mantel analysis (Figure 5) revealed a highly significant and relatively strong positive correlation between fungal communities and leaf colonization rates, further underscoring the regulatory influence of AMF on fungal communities. This result suggested a potential overlap in the ecological niches of AMF and fungal communities, though this interpretation remains speculative in the absence of direct niche characterization. Bacterial communities primarily influenced the C-H, C-O, and C=C bonds in leaves. In contrast, fungal communities predominantly affected C-O bonds, suggesting that the microorganisms in this study primarily targeted aliphatic compounds. This pattern might be attributable to the relatively short duration of decomposition. RDA (Supplementary Figure S11) further indicated that leaf colonization rate was the principal environmental factor significantly shaping both bacterial and fungal community structures, followed by the leaf C/N ratio. These results support that AMF substantially altered the microbial composition of leaves, likely through initial modification of fungal community structure, which subsequently influenced bacterial community dynamics.

We also acknowledge that the microbial community shifts observed in AMF-inoculated treatments may arise from multiple, non-mutually exclusive mechanisms. AMF hyphae may directly alter the microbial environment through the release of certain secretions and the creation of a hyphosphere niche that selectively recruits specific bacterial and fungal taxa (Zhang L. et al., 2022). However, our current experimental design does not allow us to determine exactly what substance AMF releases that causes this phenomenon. Other, AMF colonization may indirectly reshape microbial communities by modifying litter chemistry. For example, colonization may physically disrupt leaf tissue or alter C/N stoichiometry (Nuccio et al., 2013). These changes would thereby modify the resource base available to saprotrophs. We also cannot rule out the possibility that the experimental results are the result of both pathways acting together. At the same time, the AMF inoculum may have introduced other microorganisms, which could also lead to changes in the microbial community within the system. Further investigation through more carefully controlled experiments is required to confirm this.

## 5 Conclusion

AMF played a pivotal role in mediating the cycling of C, N, and P, thereby supporting both their own development and the proliferation of leaf-associated microbial communities. Through the direct colonization of litter leaves by RH, AMF actively decomposed aliphatic compounds, which accelerated the enrichment of C, N, and P within the substrate. By occupying ecological niches that would otherwise be dominated by saprophytic fungi, AMF reshaped the microbial community structure of leaves, initiating a cascade of effects that extended to bacterial populations. This process enhanced both the richness and diversity of bacterial communities, with particularly pronounced increases in the abundance of *Rhizobiales*, thereby promoting nutrient turnover and facilitating litter decomposition. However, several constraints limit the broader extrapolation of these results. The use of a single AMF, a single litter species, and a 90-day Petri-dish incubation this study a mechanistic proof-of-concept rather than a field-scale simulation. The absence of soil heterogeneity, macrofauna, leaching, and natural environmental fluctuations further restricts direct application to natural litter layers. Future research incorporating multiple AMF taxa, diverse litter chemistries, host-plant presence, longer incubations, and field validation is essential to assess the ecological generality of AMF-mediated litter decomposition and its potential implications for ecosystem carbon dynamics.

## Data Availability

The data presented in this study can be found in the NCBI (https://www.ncbi.nlm.nih.gov), accession number PRJNA1503789.
