# Supplementary material for: Arbuscular mycorrhizal fungi modulate litter decomposition dynamics through alterations in stoichiometry, chemical composition, and microbial communities
Source: Front Microbiol. 2026 Aug 31;17:1925104. doi: 10.3389/fmicb.2026.1925104 (PMC13572666; doi:10.3389/fmicb.2026.1925104)
Supplement: Supplementary file 1 [file Supplementary_file_1.DOCX]

**Supplementary Material for**

**Arbuscular Mycorrhizal Fungi Modulate Litter Decomposition Dynamics through Alterations in Stoichiometry, Chemical Composition, and Microbial Communities**

*Submitted by*

Yinli Bi ^a,b,*, †^ , Weixuan Guo ^a, †^ , Li Xiao ^a,*^ , Jianying Zhang ^c^ , Yuguang Zhang ^c^, Dongdong Wang ^b^ , Xintong Hu ^a^

^a^ Institute of Ecological Environment Restoration in Mine Areas of West China, College of Geology and Environment, Xi’an University of Science and Technology, Xi’an 710054, China

^b^ State Key Laboratory for Fine Exploration and Intelligent Development of Coal Resources, China University of Mining and Technology (Beijing), Beijing 100083, China

^c^ SDIC Hami Energy Development Co., Ltd.，Xinjiang Hami 839000, China

^*^ Corresponding authors: Yinli Bi; Li Xiao

E-mail: ylbi88@126.com (Yinli Bi) ; xiaoli047@126.com (Li Xiao)

^†^ Yinli Bi and Weixuan Guo contributed equally to this work and share first authorship.

**Tables: 2.**

**Figures: 11.**

**Contents:**

**Table S1** Basic physicochemical properties of substrate.

**Table S2** Assignment of FTIR absorption peaks for functional groups in litter

**Figure S1** Changes in spore abundance across different treatments.
**Figure S2** Changes in leaf colonization rate under different treatments.
**Figure S3** Variations in litter weight loss and decomposition rates under different treatments.
**Figure S4** (A) C/N ratio, (B) C/P ratio, and (C) N/P ratio of different treatments for litter.

**Figure S5** FTIR spectrum of litter.

**Figure S6** Proportion of primary relative peak area of functional group-associated bands in litter.

**Figure S7** AMF in litter from Amorpha fruticose.

**Figure S8** Changes over time in the following indices for litter under different treatments: (A) bacterial Chao 1, (B) bacterial Shannon, (C) bacterial Simpson, (D) fungal Chao 1, (E) fungal Shannon, and (F) fungal Simpson.

**Figure S9** Principal Coordinate Analysis (PCoA) of different treatments of litter.

**Figure S10** LEFse analysis of microorganisms in litter of different treatments.

**Figure S11** RDA analysis of physicochemical factors and (A) bacteria and (B) fungi under different treatments.

**Table S1** Basic physicochemical properties of substrate.

| TC (g/kg) | TN (g/kg) | TP (g/kg) |
| --- | --- | --- |
| 11.27±2.18 | 0.24±0.03 | 0.24±0.00 |

Note: TC: total carbon; TN: total nitrogen; TP: total phosphorus.

**Table S2** Assignment of FTIR absorption peaks for functional groups in litter (Dai et al., 2025).

| Wavenumber(cm^-1^) | Assignment |
| --- | --- |
| C-O | 1000-1300 |
| C=C | 1480-1550 |
| C=O | 1650-1750 |
| C-H | 2800-3000 |
| O-H | 3300-3400 |

Dai, W., Xiao, R., Wei, C., and Yang, F. (2025). Plant litter traits control the accumulation of mineral-associated organic carbon by influencing its molecular composition and diversity. *Soil and Tillage Research* 253**,** 106667. doi: 10.1016/j.still.2025.106667.

**Figure S1** Changes in spore abundance across different treatments.

Note: CK: non-mycorrhizal + surface-sterilized leaves, S: mycorrhizal + surface-sterilized leaves, L: non-mycorrhizal + non-surface-sterilized leaves, and SL: mycorrhizal + non-surface-sterilized leaves. Mean (n=9) values ± SE are shown. Values with different letters are significantly different at P <0.05 (Uppercase letters: different treatments at the same time, lowercase letters: different time points in the same treatment, shared letters indicate no significant difference). Subsequent figures follow the same method.

**Figure S2** Changes in leaf colonization rate under different treatments.

(A): weight loss ; (B) decomposition rates

**Figure S3** Variations in litter weight loss and decomposition rates under different treatments.

**Figure S4** (A) C/N ratio, (B) C/P ratio, and (C) N/P ratio of different treatments for litter.

Note: Leaf C/N: leaf total carbon / leaf total nitrogen; Leaf C/P: leaf total carbon / leaf total phosphorus; Leaf N/P: leaf total nitrogen / leaf total phosphorus.

(A): CK; (B): S; (C): L; (D): SL

**Figure S5** FTIR spectrum of litter.

**Figure S6** Proportion of primary relative peak area of functional group-associated bands in litter.

**Figure S7** AMF in litter from Amorpha fruticose.

**Figure S8** Changes over time in the following indices for litter under different treatments: (A) bacterial Chao 1, (B) bacterial Shannon, (C) bacterial Simpson, (D) fungal Chao 1, (E) fungal Shannon, and (F) fungal Simpson.

(A): bacterial communities; (B): fungal communities

**Figure S9** Principal Coordinate Analysis (PCoA) of different treatments of litter.

(A): bacteria; (B): fungi

**Figure S10** LEFse analysis of microorganisms in litter of different treatments.

**Figure S11** RDA analysis of physicochemical factors and (A) bacteria and (B) fungi under different treatments.
